# A first *CLN6* variant case of late infantile neuronal ceroid lipofuscinosis caused by a homozygous mutation in a boy from China: a case report

**DOI:** 10.1186/s12881-018-0690-x

**Published:** 2018-10-01

**Authors:** Guilian Sun, Fang Yao, Zhuoling Tian, Tianjiao Ma, Zhiliang Yang

**Affiliations:** Department of Pediatrics, The First Hospital of China Medical University, No. 155 Nanjing North Street, Heping District, Shenyang, 110001 Liaoning Province People’s Republic of China

**Keywords:** Neuronal ceroid lipofuscinosis, Neurodegeneration, *CLN6*, Endoplasmic reticulum, Lysosomal storage disorder

## Abstract

**Background:**

Neuronal ceroid lipofuscinosis (NCLs) are lysosomal storage disorders characterized by seizures, motor impairment, and loss of vision. Ceroid lipofuscinosis (CLN) gene mutations are the cause, but NCL cases arising from *CLN6* mutations have not been described in China to date. The CLN6 protein, which plays a role in lysosomal function, is an endoplasmic reticulum (ER) membrane protein with seven transmembrane (TM) domains. It has a cytosolic-facing amino terminal domain and a luminal-facing carboxyl terminal domain, with six loops between the TM domains.

**Case presentation:**

Here we report a case involving a Chinese boy whose suspected diagnosis was a hereditary leukoencephalopathy, based on brain MRI imaging and epilepsy symptoms, language articulation disorders, ataxia, and unstable gait. The electroencephalogram showed epileptic discharges, and the brain MRI scan showed high signal intensity adjacent to the bilateral posterior horns of the lateral ventricles on T2-weighted images, along with cerebellar atrophy. Using next-generation sequencing for the genes in a panel for hereditary leukoencephalopathies, we detected a homozygous missense point mutation c.892G > A(p.Glu298Lys) in *CLN6*, and the variant was interpreted as pathogenic on in silico analysis. Absence of this mutation was confirmed in 259 controls. Late infantile NCL and secondary epilepsy were diagnosed, and oral sodium valproate was prescribed. The epilepsy was not well controlled, however, and the other signs had not improved at the 6-month follow-up. We also analyzed the loci of 31 CLN6 missense mutations, including those previously reported and the current one. We found that 22.6% (7/31) of the mutations are in the cytoplasmic domains, about 32.2% (10/31) are in the TM domains, and about 45.2% (14/31) are in the luminal domains. These mutations were mostly located in the TM3-TM4 loop (6/31), TM1-TM2 loop (4/31), and C-terminus (4/31), with none found in the TM4-TM5 loop, TM5-TM6 loop, or TM7.

**Conclusions:**

We report the first case in China of NCL caused by a *CLN6* mutation, expanding the genotype options for NCLs. In practice, NCLs generally are not the initial suspected diagnosis for such cases. Use of a gene sequencing panel for investigating unexplained seizures or leukoencephalopathies can help confirm the diagnosis.

**Electronic supplementary material:**

The online version of this article (10.1186/s12881-018-0690-x) contains supplementary material, which is available to authorized users.

## Background

Neuronal ceroid lipofuscinosis (NCLs), usually referred to collectively as Batten disease, are a group of autosomal recessive neurodegenerative disorders characterized by seizures, progressive cognitive decline, motor impairment, and vision loss. Its prevalence is about 1:1,000,000 to 1:14,000, depending on the region [1]. The condition predominantly affects children and is incurable. The pathology for NCLs is the accumulation of autofluorescent lipopigments in various tissues because of ceroid lipofuscinosis (CLN) gene mutations, such as *CLN1*, *CLN2* (*TPP1*), *CLN5*, *CLN10*, and *CLN13*, which all encode lysosomal enzymes/proteins; *CLN3*, *CLN7* (*MFSD8*), and *CLN12*, which encode lysosomal membrane proteins; *CLN4* and *CLN14*, encoding cytosolic proteins; *CLN6* and *CLN8*, encoding endoplasmic reticulum (ER) membrane proteins; *CLN11*, involved in the secretory pathway; and *CLN9*, which remains unidentified [2]. NCLs have been broadly divided into four subgroups based on age of onset: infantile, late infantile, juvenile, and adult. Late infantile neuronal ceroid lipofuscinosis (LINCLs) occur between ages 2 and 4 years [3].

In the NCL mutation database, cases associated with *CLN1*, *CLN2*, and *CLN5* mutations have been collected from China (http://www.ucl.ac.uk/ncl/mutation.shtml). However, those associated with NCLs (primarily LINCLs) caused by *CLN6* mutations have been collected from Europe, America, and some Asian countries, such as India, Pakistan, and Japan, but not from China (http://www.ucl.ac.uk/ncl/CLN6patienttable.htm). CLN6, which plays a role in lysosomal function [4, 5], is an ER membrane protein with seven transmembrane (TM) domains, an amino terminal domain facing the cytosol, a carboxyl terminal domain facing the ER lumina, and six loops connecting the TM domains. The TM domains are TM1 to TM7 from the N-terminus to the C-terminus.

Here we report the first case in China to involve genetically confirmed LINCL tracing to a homozygous *CLN6* mutation. The parents of the boy are heterozygous without any signs of NCL.

## Case presentation

**Fig. 1:**
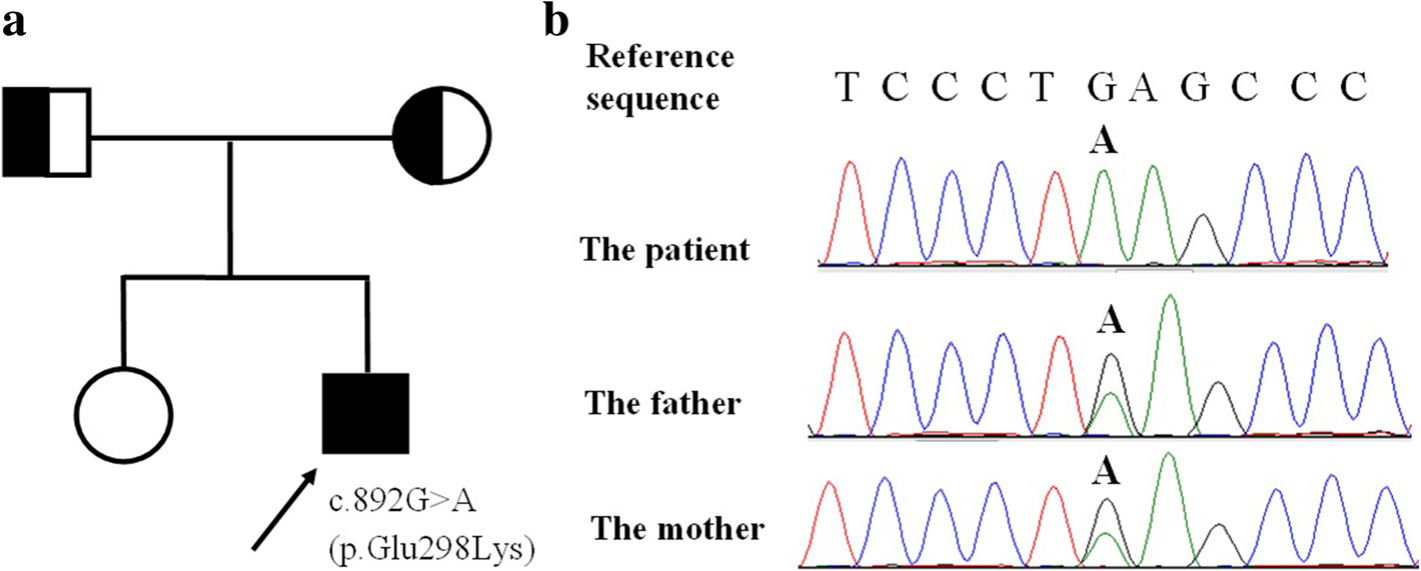
The family pedigree showing the mutations detected in *CLN6*. **a** The pedigree of the family with late infantile neuronal ceroid lipofuscinosis (LINCL). The arrow indicates the proband; his parents have no signs of LINCL. **b** The mutations detected in the family. The proband is homozygous, while the parents are heterozygous

The proband (Fig. 1a) was a 5-year-old Chinese boy admitted to the hospital with a chief complaint of language articulation disorders for 1.5 years, uncoordinated movements for half a year, and repeated episodes of seizures for one week. A stutter was noted without obvious causes one and a half years before, and he had gradually developed unclear enunciation, clumsy speech, and slow response. Within the previous 6 months, he began to have uncoordinated movements, such as ataxia and instable gait. Three episodes of generalized tonic–clonic seizures had occurred in the week just preceding the clinic visit; every episode lasted for about 1 min and occurred about once every 2 days. He achieved appropriate developmental milestones before age 3.5 years and was born at full-term by vaginal delivery without any complications. There was no exposure to alcohol or medications during the pregnancy, and Apgar scores were 10 and 10 at 1 and 5 min, respectively. His parents had no known consanguinity, and both they and an older sister were healthy.

**Fig. 2:**
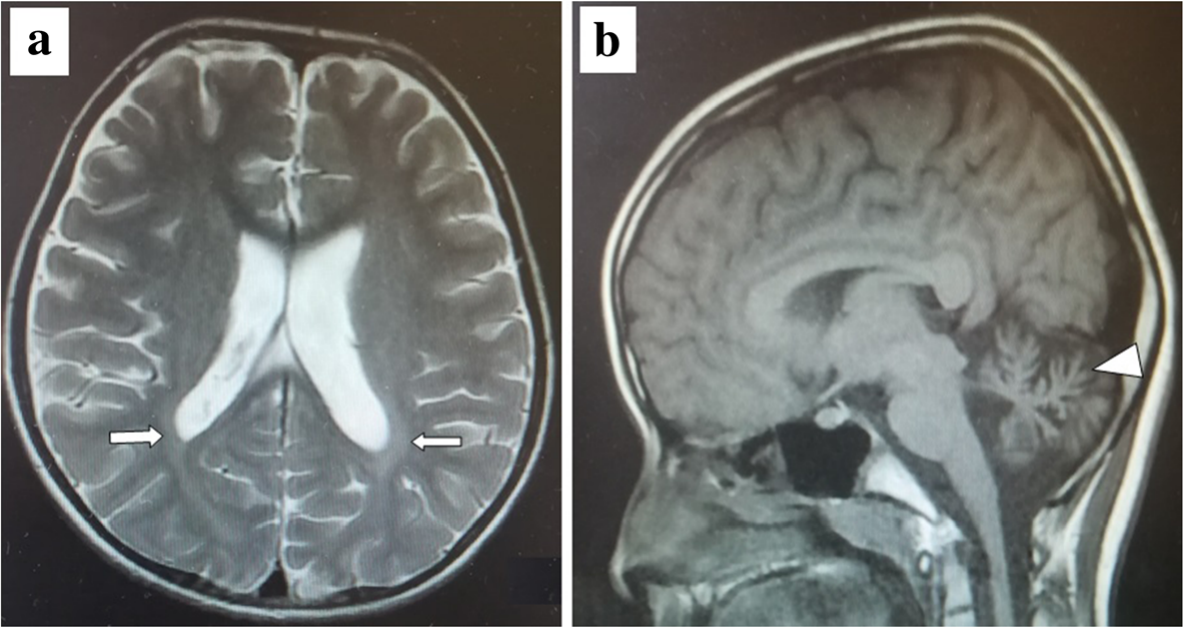
The brain MRI images. **a** T2-weighted brain MRI image shows high signals adjacent to the bilateral posterior horns of the lateral ventricles (arrows). **b** T1-weighted brain MRI image shows broadened cerebellar fissures (arrowhead)

On physical examination, length, weight, and head circumference were normal for age, and he was conscious and showed normal muscle strength and muscle tone. Patellar reflex and Achilles tendon reflex were normal, and Babinski signs were negative. He was inarticulate and responded to questions slowly. He could not complete the hand-alternating movement test, heel–knee–tibia test, or finger–nose test because of poor cooperation with the instructions. The intelligence quotient value, measured by combined Raven’s test, was 80 (a medium level). The tests for blood lactic acid, homocysteine, ammonia, ceruloplasmin, and liver and kidney function were normal. Tests for antibodies for autoimmune encephalitis in cerebrospinal fluid and blood were negative. The screening for genetic metabolic diseases in blood and urine showed no obvious abnormalities. An electroencephalogram (EEG) showed multiple spikes and slow-wave discharges bilaterally. A brain MRI scan showed high hyperintensities adjacent to the bilateral posterior horns of the lateral ventricles on T2-weighted images and broadened cerebellar fissures (Fig. 2). Leukoencephalopathy was considered, and genetic analysis for hereditary leukoencephalopathies was recommended.

With written consent from his parents, peripheral blood samples were collected from the proband and his parents. DNA was extracted using the Puregene Extraction Kit (Qiagen, USA). The NextSeq500 sequencer (Illumina Inc., USA) was used to screen the exons in the genes related to hereditary leukoencephalopathies. The genes in the panel are listed in Additional file 1. The data obtained were analyzed using accompanying software, and the variants were called according to the protocol. The variants were interpreted according to the guidelines from the American College of Medical Genetics and Genomics and patient phenotype [6]. Direct sequencing validated the detected missense mutations. Direct sequencing was performed on DNA from the proband and his parents using the ABI3500sequencer (Life Technology, USA), and the samples were subjected to sequence analysis using Sequence Scanner v1.0 (Applied Biosystems, USA). The matched Chinese controls were obtained from the Shenyang Kingmed for Clinical Laboratory (Shenyang, China). The sequencing procedure and mutation validation were performed by Shenyang Kingmed for Clinical Laboratory (Shenyang, China), which provides third party inspection services.

The possible effects of the mutations on protein function were analyzed using the Polymorphism Phenotyping v2 (PolyPhen-2) prediction tool (http://genetics.bwh.harvard.edu/pph2/dbsearch.shtml), SIFT (http://sift.jcvi.org/www/SIFT_enst_submit.html), and MutationTaster (http://www.mutationtaster.org/index.html).

The genetic analysis showed that the proband had a homozygous missense point mutation c.892G > A(p.Glu298Lys) (reference sequence: NM_017882.2) in exon 7 in *CLN6* and that both his parents were heterozygous for the mutation (Fig. 1b). The mutation was not detected in 259 control subjects. LINCL was diagnosed, and a visual test was performed and showed no obvious abnormalities. PolyPhen-2, SIFT, and MutationTaster analysis suggested that the mutation would negatively affect protein function (Table 1). The mutation in the patient is the first reported, and we can define it as recessive.

**Table 1:** Functional evaluation of the *CLN6* mutations detected in the family of a Chinese boy with late infantile neuronal ceroid lipofuscinosis

Base change	Exon number	Amino acid change	PolyPhen-2 analysis	SIFT analysis	MutationTaster analysis
c.892G > A	7	p.Glu298Lys	Probably damaging	Damaging	Disease causing

Because of the history of seizures and the EEG results, the patient was diagnosed with epilepsy, and oral sodium valproate (VPA) was ordered. VPA administration was initiated at 15 mg/kg per day, administered in two doses, increasing to about 25 mg/kg per day in 2 weeks. The blood concentration range was 56–78 μg/ml over 6 months. At the 6-month follow-up, the episodes were decreased to about once a month, articulation disorders and uncoordinated movements persisted, and visual loss was not detected.

At the locus of *CLN6*, 31 missense mutations including the reported ones and ours were analyzed; 22.6% (7/31) were located in the cytoplasmic domains, 32.2% (10/31) in the TM domains, and 45.2% (14/31) in the luminal domains of the protein. Regarding each domain of the protein, the mutations were mostly located in the TM3-TM4 loop (6/31), TM1-TM2 loop (4/31), and C-terminus (4/31), and no mutations were reported in the TM4-TM5 loop, TM5-TM6 loop, and TM7 domain (Table 2).

**Table 2:** Distribution of missense mutations in CLN6 according to the position

Positions (*n*,%)	Domains	Missense mutations (*n*)
Cytosol domains (7, 22.6%)	N-terminus	3(p.Arg5Trp, p.Ala12Thr, p.Gly17Ser)
	TM2-TM3 loop	2(p.Arg103Trp, p.Ser104Phe)
	TM4-TM5 loop	0
	TM6-TM7 loop	2(p.Arg252His, p.Gly259Val)
TM domains (10, 32.2%)	TM1	2(p.Arg62His, p.Arg62Cys)
	TM2	1(p.Asn90Lys)
	TM3	2(p.(Ile119_Phe120insIle), p.Gly123Asp)
	TM4	1(p.Phe186Ser)
	TM5	2(p.Tyr221Ser, p.Tyr221Cys)
	TM6	2(p.Phe234Leu, p.Met241Thr)
	TM7	0
ER luminal domains (14, 45.2%)	TM1-TM2 loop	4(p.Pro70Leu, p.Glu72Gln, p.Asp82His, p.Asp82Val)
	TM3-TM4 loop	6(p.Arg136Cys, p.Arg149Cys, p.Pro159Leu, p.Leu162Arg, p.Leu169Pro, p.Tyr172Leu)
	TM5-TM6 loop	0
	C-terminus	4(p.Pro297Thr, p.Glu298Lys, p.Pro299Leu, p.Trp300Arg)
Total	–	31

## Discussion and conclusions

Lysosomal storage disorders (LSDs) are usually early-onset, severe, and life-limiting. NCLs are a group of LSDs, with symptoms mainly involving the nervous system, and NCLs caused by *CLN6* mutations were originally identified in a patient from Costa Rica [7]. Clinical symptoms caused by *CLN6* mutations are nonspecific; seizures and motor difficulties usually present early, followed by speech impairments, myoclonus, ataxia, mental regression, and vision loss [8]. Brain T2-weighted MRI images show high signal intensity in the periventricular white matter with some cerebellar atrophy [9]. In previous reports, visual loss appears between ages 3 and 8 years and rapidly progresses to blindness [10]. In our case, language articulation disorders and uncoordinated movements were the initial signs, but unfortunately such signs went unrecognized during earlier clinical visits and were not addressed until the repeated seizure episodes. Ophthalmology testing showed no obvious visual loss, and the vision continues to be checked periodically.

We reviewed all *CLN6* missense mutations in NCLs and found that the missense mutations distributed mostly in luminal domains (45.2%), followed by TM domains (32.2%), and then cytoplasmic domains (22.6%). Mutations occurred relatively more often in the TM3-TM4 loop, TM1-TM2 loop, and C-terminus, and no reported mutations have been localized to date in the TM4-TM5 loop, TM5-TM6 loop, or TM7. Our patient had the homozygous mutation located in the C-terminus, which is a relative hotspot for CLN6 mutations. Neither parents have NCLs, although they both are heterozygous for mutations that were predicted to be disease-causing on in silico analysis. Neither the homozygous nor the heterozygous mutation was identified in the Chinese matched controls. The mutation in the family is autosomal recessive according to this information, and the homozygous status is considered to be the cause of the patients symptoms.

To date, how mutations in CLN6 cause disease is still unclear. CLN6 is involved in the pathology with functioning in endocytosis of lysosomal proteins, in selective transport of lipids and proteins associated with the function and acidification of the lysosome, and in autophagy [11]. These mechanisms may reasonably explain the phenotype difference associated with *CLN6* mutations and those tied to other mutations. The age of onset with *CLN6* mutations shows a bimodal mode in infantile and adult versions of the disease, with patients tending to be older than with other gene mutations [12]. The signs and symptoms associated with *CLN6* mutations can also vary according to the different loci. Some severe signs may arise earlier, but accumulation of relatively mild damage requires time so that the progression is less rapid. This distinction may explain the nonoverlapping ages of onset and various phenotypes.

ER plays a critical role for normal cell function. Alteration of ER homeostasis can lead accumulation of misfolded protein and activate the unfolded protein response to induce some diseases [13]. The abnormalities of ER caused by CLN6 mutations may decrease ER homeostasis and then affect lysosomal function. Increased biometal levels such as zinc and copper have been observed in CLN6 disease, and biometal dyshomeostasis can interfere with protein folding and cause activation of ER stress with subsequent neuron apoptosis [13].

No etiologic or disease-specific therapeutic approaches are currently available for NCLs, which can be treated only symptomatically. In this case, the epilepsy was not well controlled by oral VPA at the 6-month follow-up, and one more antiepileptic was considered.

In conclusion, NCLs usually are not the initial suspicion for such cases in practice, not only because of low disease awareness and nonspecific clinical presentation but also because of limited access to diagnostic approaches in some regions. The use of whole exome/genome sequencing or some gene panel for investigating unexplained seizures or leukoencephalopathies or inherited metabolic disorders can be quite helpful for diagnosis and differential diagnosis. Although no effective therapies for NCLs are available, early diagnosis can allow for optimization of patient care.

## Additional file

Additional file 1: The genes in the used panel for inherited leukoencephalopathy. (DOC 54 kb)

## Abbreviations

CLN gene: Ceroid lipofuscinosis gene
ER: Endoplasmic Reticulum
LINCLs: Late infantile neuronal ceroid lipofuscinosis
LSDs: Lysosomal storage disorders
NCLs: Neuronal ceroid lipofuscinosis
TM Domains: Transmembrane domains
VPA: Sodium valproate
