# Supplementary material for: A first CLN6 variant case of late infantile neuronal ceroid lipofuscinosis caused by a homozygous mutation in a boy from China: a case report
Source: BMC Med Genet. 2018 Oct 1;19:177. doi: 10.1186/s12881-018-0690-x (PMC6167792; doi:10.1186/s12881-018-0690-x)
Supplement: Supplementary file 1 — The genes in the used panel for inherited leukoencephalopathy. (DOC 54 kb) [file 12881_2018_690_MOESM1_ESM.doc]

Additional file: The genes in the used panel for inherited leukoencephalopathy.
ABCD1, ARSA, CSF1R, EIF2B2, GJC2, PEX1, POLR3A, POLR3B, PSAP, TREX1, EIF2B1, EIF2B3, EIF2B4, EIF2B5, PLP1, GFAP, ASPA, MLC1, PPT1/CLN1, TPP1/CLN2, CLN3, CLN5, CLN6, MFSD8/CLN7, CLN8, CLN10/CTSD, CLCN6, HEPACAM, CYP27A1, GBE1, RNASEH2A, RNASEH2B, RNASEH2C, SAMHD1, ADAR, LMNB1, SLC17A5, FUCA1, FAM126A, GALC, L2HGDH, DARS2, EARS2, SUMF1, GJA1, RNASET2, HSD17B4, ACOX1, SCP2, ALDH3A2, SOX10, MFSD8, HSPD1, MPV17, PEX10, PEX13, PEX5, PEX6, POLR1C, SDHA, SDHB, TMEM187
